# MicroRNA-451b Participates in Coronary Heart Disease By Targeting VEGFA

**DOI:** 10.1515/med-2020-0001

**Published:** 2019-12-26

**Authors:** Jie Lin, Jun Jiang, Ruifang Zhou, Xiaojie Li, Jun Ye

**Affiliations:** 1 Translational Medicine Center, Taizhou People’s Hospital, No. 399 Hailing Road, Taizhou 225300, China; 2 Department of Cardiology, Taizhou People’s Hospital, Taizhou 225300, China; 3 Taizhou Polytechnic College, Taizhou 225300, China

**Keywords:** Coronary heart disease, miR-451, VEGFA, HUVECs, PI3K-Akt-mTOR pathway

## Abstract

Coronary artery disease (CAD) is one of the main causes of hospitalization worldwide and has high morbidity. MicroRNAs (miRNAs) play an important role in the pathogenesis of cardiovascular diseases. miR-451 is a special miRNA that is involved in many cancers’ development. At present, there is no research about miR-451 in coronary heart disease. In this study, we aimed to identify the action mechanism of miR-451 in coronary heart disease and human umbilical vein endothelial cells (HUVECs). In this study, we found that miR-451 is up-regulated in the peripheral blood of patients with coronary heart disease. Moreover, TargetScan and dual-luciferase reporter gene assay results showed that VEGFA is a direct target gene of miR-451. C (CCK-8) and flow cytometry assay results showed that miR-451 mimic significantly inhibits cell proliferation and promotes apoptosis in HUVECs. Moreover, we found that the role of miR-451 in HUVECs is associated with the PI3K-Akt-mTOR pathway. Taken together, the data indicates that miR-451 might be a novel bio-marker for coronary heart disease.

## 1 Introduction

Coronary artery disease (CAD) is typically caused by the development of atherosclerotic lesions and results in myocardial ischemia [1, 2]. CAD is related with inflammation and thrombosis, which can cause luminal stenosis or occlusion [3]. Although tremendous progress in the treatment of CAD has been made, it remains one of the leading causes of morbidity and mortality all over the world, causing major social and economic burdens [4, 5].

So far, many epidemiological studies have demonstrated several risk factors for CAD, including dyslipidemia, diabetes, high blood pressure, smoking, and diet [6, 7, 8]. A growing body of evidence suggest that abnormal changes in the expression of some genes in CAD play a crucial role in the pathogenesis of atherosclerosis [9, 10]. MicroRNAs, also known as miRNAs or small RNAs, are short-chain non-coding RNAs with regulatory functions that are widely found in plants and animals [11]. miRNAs can regulate protein translation by either complete or incomplete pairing with the target gene, or by inhibiting expression of downstream target proteins [12, 13]. miRNAs are involved in a variety of physiological processes including cell proliferation, apoptosis, signal transduction, differentiation, metabolism and hormone secretion, and have the potential to maintain embryonic stem cells [13]. mRNAs could regulate the growth and development of the human body and adapt to the environment. Studies have shown that miRNAs are related to the development of cancer [14]. Currently, a large amount of research on miRNAs focus on their effects on tumorigenesis, development, invasion, metastasis and other biological characteristics [15].

miR-451 is a tumor suppressor gene that plays a role in tumor suppression in a variety of cancers [16]. A previous study has found that miR-451 could inhibit hepatic tumor angiogenesis, revealing that it may be involved in the formation of blood vessels [17].

Functional damage of endothelial cells leads to atherosclerosis, but the specific function and mechanism of miR-451 in human umbilical vein endothelial cells remains unclear. Bioinformatics methods indicated that VEGFA was a direct target of miR-451b. Therefore, the aim of this study was to investigate the role of miR-451b in coronary heart disease (CHD) through exploring the role of miR-451b in human umbilical vein endothelial cells.

## 2 Materials and methods

### 2.1 Clinical samples

30 samples of peripheral blood of patients with coronary heart disease (age range, 46-59 years; male/female, 15/15), and 30 samples of peripheral blood of healthy control volunteer (age range, 45-58 years; male/female: 15/15) without coronary heart disease were collected. This study was approved by the Ethics Committee of Taizhou People’s Hospital. Written inform consent was obtained from every patient.

### 2.2 Detection of serum HDL, LDL, TC and TG levels

2 ml blood samples from each participant in fasting state were collected. Serum was collected by centrifuging at 3000×g for 15 min. ELISA assays were performed to detect the serum levels of HDL (Abcam), LDL (Abcam), TC (Abcam) and TG (Abcam) using kits according to the manufacturer’s protocol.

### 2.3 Cell culture

HUVECs were purchased from Shanghai Institute of Life Sciences, Chinese Academy of Sciences. HUVECs were cultured in routine medium 199 (Gibco) with 15% fetal bovine serum (FBS; Gibco; Thermo Fisher Scientific, Inc.), and endothelial cell growth supplement (Sigma) plus epidermal growth factor (EGF 10 ng/ml) in a 5% CO_2_ humidified atmosphere at 37°C.

### 2.4 Cell transfection

HUVECs (5.0x10^4^ cells per well) were transfected with negative control miR-451b mimics (mimics NC) (Guangzhou Ribobio Co., Ltd., Guangzhou, China), miR-451b mimics (Guangzhou Ribobio Co., Ltd., Guangzhou, China), or miR-451b mimics +VEGFA plasmid (Santa Cruz, USA) for 48 h at 37°C using Lipofectamine^®^ 2000 (Invitrogen; Thermo Fisher Scientific, Inc.) according to the manufacturer’s instructions. 48 h after transfection, the transfection efficiency was detected using qRT-PCR.

### 2.5 Quantitative reverse transcription-polymerase chain reaction (qRT-PCR)

Total RNA was extracted via Trizol reagents (Invitrogen; Thermo Fisher Scientific, Inc.) from cells according to the manufacturer’s protocol. We detected the concentration of total RNA via a NanoDrop Spectrophotometer. cDNA was synthesized by using the PrimeScript Reverse Transcriptase Reagent kit (Takara Biotechnology Co., Ltd.). Then, we performed real-time fluorescent quantitative PCR to detect the expression level of miR-451b using SYBR Premix Ex Taq kit (Takara Biotechnology Co., Ltd.) according to the manufacturer’s protocol. The relative expression levels of miR-451b were compared between samples using U6 as control. The primers used were as follows:

U6, forward 5’-GCTTCGGCAGCACATATACTAAAAT-3’,

reverse 5’-CGCTTCACGAATTTGCGTGTCAT-3’;

miR-451b, forward 5’- CTGGAGAAACCGTTACCATTAC-3’;

reverse 5’-GTGCAGGGTCCGAGGT-3’. Relative expression levels of genes were calculated by the 2-ΔΔCt method [18]. All experiments were performed in triplicate.

### 2.6 Western blotting

Cells were solubilized in lysing buffer and proteins were extracted. The proteins were separated by 12% sodium dodecyl sulfate (SDS)-polyacrylamide gel electrophoresis (PAGE), followed by transfer to polyvinylidenefluoride (PVDF) membrane. After blocking with 5% non-fat milk, the membrane was probed with primary antibodies: VEGFA (cat. no. Ab52917; 1:1,000; Abcam), PI3K (cat. no. 4257; 1:1,000; Cell Signaling Technology, Inc.), p-PI3K (cat. no. 17366; 1:1,000; Cell Signaling Technology, Inc.), AKT (cat. no. 4691; 1:1,000; Cell Signaling Technology, Inc.), p-AKT (cat. no. 4060; 1:1,000; Cell Signaling Technology, Inc.), mTOR (cat. no. 2983; 1:1,000; Cell Signaling Technology, Inc.), p-mTOR (cat. no. 5536; 1:1,000; Cell Signaling Technology, Inc.), and GAPDH (cat. no. 5174; 1:1,000; Cell Signaling Technology, Inc.). Then, HRP-conjugated secondary antibody (cat. no. 7074; 1:1,000; Cell Signaling Technology, Inc.) was added and membranes were incubated for a further 2 h at room temperature. GAPDH was used as the protein loading control. Signals were detected with an ECL system (Merck) according to the manufacturer’s instructions.

### 2.7 CCK-8 assays

We performed CCK8 assays to detect cell viability. Briefly, HUVECs were seeded onto 96-well plates at the density of 5x10^3^ cells/well and cultured for 12, 24 or 48 h, then 10 μl CCK-8 (Sigma-Aldrich, Merck KGaA) was added to medium. After incubation for 4 h, the absorbance was measured at a wavelength of 450 nm using a micro-plate reader.

### 2.8 Dual luciferase reporter assays

TargetScan (http://www.targetscan.org/vert_72/) was used to predict the potential targets of miR-451b, and the binding sites between miR-451b and VEGFA were observed. Then to confirm the binding sites between miR-451b and VEGFA, dual luciferase reporter assays were carried out. For the dual luciferase reporter assay, miR-451b mimics, the negative control of miR-451b mimics (NC), and the wild-type (WT) or mutant (Mutant) 3’-UTR of VEGFA were co-transfected into HUVECs in a 24-well plate for 48 h using Lipofectamine 2000 (Invitrogen). Luciferase activity was measured using the Dual-Luciferase Reporter Assay System (Promega, Madison, WI, USA) according to the manufacturer’s instructions. Renilla luciferase was used for normalization. Each sample was performed three times.

### 2.9 Flow cytometry assay

Cell apoptosis was detected by using Annexin V-fluorescein isothiocyanate (FITC) Apoptosis Detection Kit I (BD Bioscience, San Diego, CA, USA) according to the manufacturer’s protocol. Cells were harvested, centrifuged, and re-suspended in 100 μl of FITC-binding buffer. Approximately 5 μl of ready-to-use Annexin V-FITC (BD Bioscience) and 5 μl of propidium iodide (PI) were added to the mixture. Cells were incubated for 30 min in the dark at room temperature. Annexin V-FITC and PI fluorescence were assessed by BD FACSCalibur flow cytometer (BD Technologies). Data were analyzed using FlowJo software (version 7.6.1; FlowJo LLC, Ashland, OR, USA).

### 2.10 Statistical analysis

Experiments were repeated in triplicate. SPSS 19.0 software (SPSS, Inc, Chicago, IL, USA) was used for data analysis. Comparisons between groups were analyzed using Student’s t test or one-way analysis of variance analysis followed by Tukey’s post-hoc test. The data were expressed as the mean ± standard deviation (SD), and p<0.05 was considered as significant.

## 3 Results

### 3.1 The expression level of miR-451b in the serum of CHD patients

**Figure 1:**
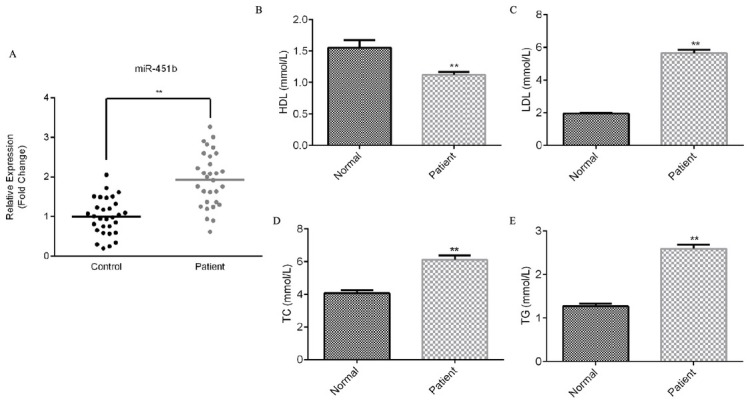
miR-451b is up-regulated in the serum of patients with coronary heart disease. (A) qRT-PCR assay detected the relative expression of miR-451. (B-E) ELISA assay detected the level of high density lipoprotein, low density lipoprotein, serum total cholesterol, and triglyceride in the serum of patients with coronary heart disease. **p<0.01.

To detect the expression of miR-451b in the serum of CHD patients, and to explore the correlation between biochemical markers such as high-density lipoprotein, low-density lipoprotein, serum total cholesterol and triglyceride in peripheral blood and miR-451b, we collected 30 samples of peripheral blood of patients with coronary heart disease, and 30 samples of peripheral blood of healthy patients without coronary heart disease. We performed qRT-PCR to detect the relative expression of miR-451b in the serum of CHD patients and in the serum of healthy volunteers. The results showed that miR-451b was highly expressed in the serum of patients with coronary heart disease (Figure 1A). Compared with the healthy volunteers, high density lipoprotein (HDL) was down-regulated, low density lipoprotein (LDL), serum total cholesterol (TC), and triglyceride (TG) were significantly up-regulated in the serum of patients with coronary heart disease (Figure 1B-E).

### 3.2 VEGFA is a direct target of miR-451b

**Figure 2:**
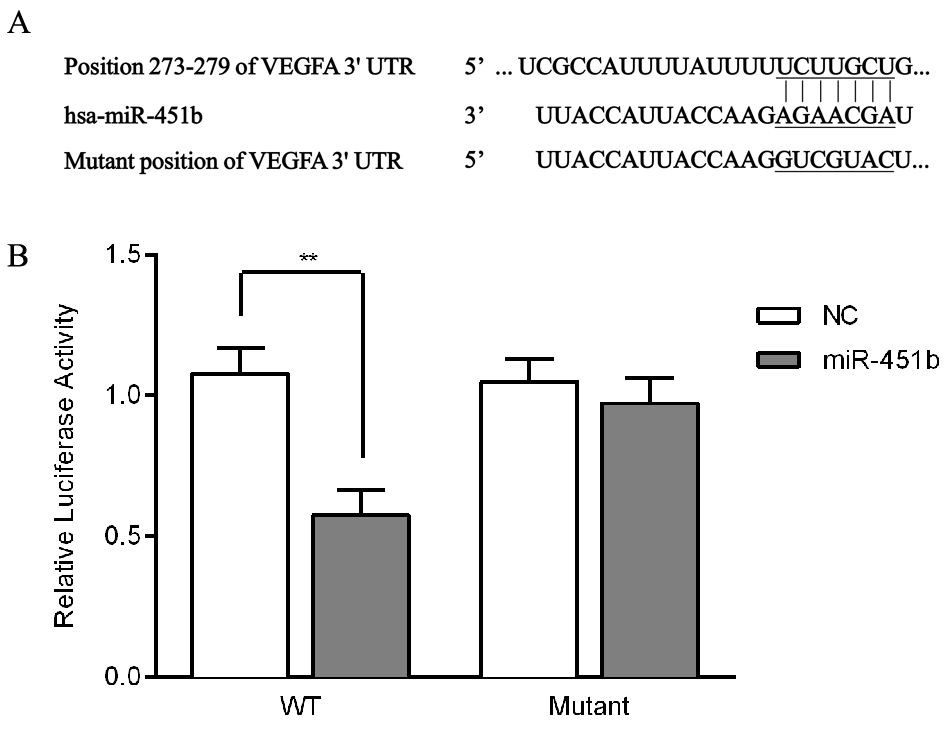
VEGFA is a direct target of miR-451b. (A) The binding sites between miR-451b and the 3’-UTR of VEGFA. (B) Dual-luciferase reporter assay was performed to verify the binding sites between miR-451b and the 3’-UTR of VEGFA. **p<0.01.

We performed bioinformatics (TargetScan) to predict the potential targets of miR-451b. Among these genes, VEGFA was identified as a potential target gene based on the predicted binding sites of miR-451b at its 3’UTR (Figure 2A). To further verify the relationship between miR-451b and VEGFA, dual-luciferase reporter gene assay was used. The results showed that miR-451b decreased the activity of the luciferase reporter fused to the 3’-UTR-WT of VEGFA but did not inhibit that of the reporter fused to the MUT version (Figure 2B). Taken together, these results showed that VEGFA is a direct target gene of miR-451b.

### 3.3 Effect of miR-451b on proliferation and apoptosis in HUVECs

**Figure 3:**
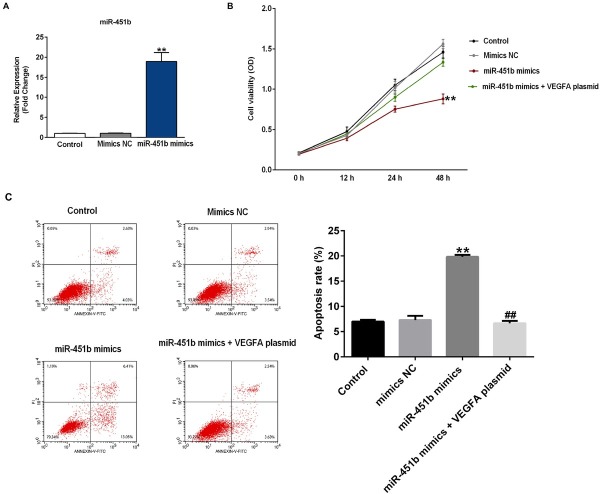
miR-451b inhibits cell proliferation and promotes cell apoptosis in HUVECs. (A) qRT-PCR assay detected the relative expression of miR-451b when HUVECs were transfected with miR-451b mimics or NC mimics for 48 h. (B) CCK-8 assays were carried out to determine cell viability when the HUVECs were transfected with miR-451b mimics, or miR-451b mimics+VEGFA plasmid for 48 h. (C) Flow cytometry analysis analyzed the percentage of apoptotic cells when HUVECs were transfected with miR-451b mimics, or miR-451b mimics+VEGFA plasmid for 48 h. The cell apoptosis rate was calculated and presented. **p<0.01 vs. mimics NC; ##p<0.01 vs. miR-451b mimics.

To examine the transfection efficiency of miR-451b in HUVECs, we performed qRT-PCR to detect the expression level of miR-451b. The results indicated that compared with the control group, the miR-451b mimic significantly increased the expression of miR-451b (Figure 3A) in HUVECs. In order to shed light on the function of miR-451b in HUVECs, we first investigated the effect of miR-451b on the proliferation of HUVECs. CCK-8 assay results indicated that compared with the control, miR-451b mimics could significantly inhibit the cell viability of HUVECs, while this effect was reversed by VEGFA plasmid (Figure 3B). To further determine whether miR-451b could regulate apoptosis of HUVECs, we performed flow cytometry to examine cell apoptosis. Flow cytometry analysis showed that miR-451b significantly promoted cell apoptosis; while when HUVECs were co-transfected with miR-451b mimics and VEGFA plasmid, cell apoptosis decreased (Figure 3C).

### 3.4 Effect of miR-451b on PI3K-Akt-mTOR pathway

**Figure 4:**
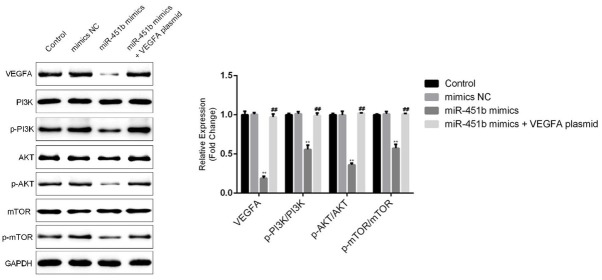
miR-451b mimics inhibit the PI3K-Akt-mTOR pathway in HUVECs. After cell transfection, western blot analysis was used to detect the protein expression levels of VEGFA, PI3K, p-PI3K, AKT, p-AKT, mTOR, and p-mTOR in HUVECs. The protein levels were quantified. **p<0.01 vs. mimics NC; ##p<0.01 vs. miR-451b mimics.

To explore the underlying mechanism of the role of miR-451b in HUVECs, we investigated the expression levels of related proteins in the PI3K-Akt-mTOR pathway. We found that miR-451b mimics decreased the protein expression of VEGFA. Western blot assays showed that miR-451b mimics decreased the protein expression levels of p-PI3K, p-AKT, p-mTOR in HUVECs. However, all these effects were reversed by the VEGFA plasmid (Figure 4).

## 4 Discussion

In this study, we explored the expression level and underlying mechanism of miR-451b in coronary heart disease and endothelial cells *in vitro*. In brief, miR-451b was up-regulated in the serum of patients with coronary heart disease. Integrative bioinformatics prediction and dual-luciferase reporter assays showed that VEGFA was a target gene of miR-451b. In addition, miR-451b mimics inhibited proliferation of HUVECs and promoted cell apoptosis. VEGFA plasmid attenuated the inhibitory effect of miR-451b on HUVECs. Finally, we also found that miR-451b function in HUVECs was associated with the PI3K-Akt-mTOR pathway.

As our understanding of CAD has evolved from a focal to a systemic disease, systemic approaches to identify vulnerable patients have become preferable to the identification of local vulnerable plaques or myocardial damage [19]. Previousresearch demonstrated that miR-451 had potential activity in various cancer cells [20, 21, 22, 23]. It has been reported that miR-451 is down-regulated in human cancer and it is known as a potential tumor suppressor. It has been reported that miR-19a/19b, miR-20a, miR-26, miR-106 and miR-451 are up-regulated in vulnerable CAD patients compared to patients with stable angina or with non-cardiac chest pain [19]. Sondermeijer et al. indicated that miR-451 is enriched in the blood platelets of premature CAD patients [24].

In our study, we found that miR-451b was highly expressed in the serum of patients with coronary heart disease. However, the function and mechanism of miR-451b in human umbilical vein endothelial cells are still unclear. Therefore, in this study, we aimed to explore the role of miR-451 in human umbilical vein endothelial cells. Liu et al. reported that IL-6R is the direct target gene of miR-451. miR-451 inhibits the growth of hepatocellular carcinoma by targeting the IL-6R-STAT3 pathway [17]. Yang et al. indicated that miR-451 suppresses glioma cell proliferation and invasion by targeting CAB39 [25]. In our study, VEGFA was identified as the target of miR-451b. Vascular endothelial growth factor (VEGF) is the most important factor for inducing endothelial cell proliferation, and angiogenesis in tumor tissues is directly related to VEGF. VEGF consists of five family members, namely VEGFA, VEGFB, VEGFC, VEGFD, and VEGFE. VEGF binds to its receptors to induce its biological effects [26].

In the present study, we found that miR-451b mimics could inhibit cell proliferation and increase cell apoptosis in HUVECs. However, the VEGFA plasmid reduced the inhibitory effect. We also demonstrated that miR-451b was related to the PI3K-Akt-mTOR pathway. Similarly, in a previous study, miRNA-451 has been found to inhibit the PI3K/AKT signaling pathway in glioma cells and directly influenced the biological behavior of glioma cells. Recent studies also demonstrated the role of miR-451 in the modulation of pro-inflammatory cytokine production (e.g., macrophage migration inhibitory factor) and the PI3K/AKT pathway [24, 27, 28].

In conclusion, miR-451b might modulate HUVEC proliferation and apoptosis through affecting the PI3K-Akt-mTOR signaling pathway by altering the expression of VEGFA, and thus participating in the occurrence and development of CHD. However, this was only an *in vitro* study of the role of miR-451b in CHD. The current study did not conduct an *in vivo* study of the effects of miR-451b in CHD. This was a limitation of our study and we will further study this in the future.
